# The Associations Between Loneliness, Hopelessness, and Self-control and Internet Gaming Disorder Among University Students Who Were Men Who Have Sex With Men: Cross-sectional Mediation Study

**DOI:** 10.2196/43532

**Published:** 2023-01-17

**Authors:** Yanqiu Yu, Vivian W I Fong, Joyce Hoi-Yuk Ng, Zixin Wang, Xiaobing Tian, Joseph T F Lau

**Affiliations:** 1 Department of Preventive Medicine and Health Education School of Public Health Fudan University Shanghai China; 2 Key Laboratory of Public Health Safety Fudan University Shanghai China; 3 Center for Health Behaviours Research Jockey Club School of Public Health and Primary Care Chinese University of Hong Kong Hong Kong Hong Kong; 4 Department of Epidemiology and Biostatistics School of Public Health North Sichuan Medical College Nanchong China; 5 Zhejiang Provincial Clinical Research Center for Mental Disorders The Affiliated Wenzhou Kangning Hospital Wenzhou Medical University Wenzhou China; 6 School of Mental Health Wenzhou Medical University Wenzhou China; 7 School of Public Health Zhejiang University Hangzhou China

**Keywords:** men who have sex with men, internet gaming disorder, self-control, loneliness, hopelessness, structural equation modeling

## Abstract

**Background:**

The minority stress model postulates that men who have sex with men (MSM) often encounter multiple stressors because of their sexual minority status, which may lead to psychological problems and maladaptive coping such as addictive behaviors (eg, internet gaming disorder [IGD]). It was hypothesized that hopelessness and loneliness would be associated with IGD via self-control among MSM.

**Objective:**

This study investigated the prevalence of IGD and its associations with variables related to minority stress (loneliness and hopelessness) among MSM who were university students. Mediation involving such associations via self-control was also explored.

**Methods:**

With informed consent, 305 MSM attending universities in Sichuan, China participated in the study. The validated *Diagnostic and Statistical Manual of Mental Disorders* (Fifth Edition) checklist was used to assess IGD. Multivariable logistic regression adjusted for background factors and structural equation modeling were conducted.

**Results:**

The prevalence of IGD was 12.8% (n=39). Logistic regression found that IGD was positively associated with hopelessness and loneliness, and negatively associated with self-control. The structural equation modeling identified three significant paths between hopelessness/loneliness and IGD: (1) hopelessness → lower self-control → higher IGD (full mediation), (2) loneliness → lower self-control → higher IGD (partial mediation: effect size of 28%), and (3) a direct effect from loneliness to IGD.

**Conclusions:**

IGD was prevalent among young MSM and warrants interventions that may try to reduce the level of psychosocial problems such as loneliness and hopelessness and improve self-control. According to the socioecological model, the promotion of social acceptance and reduction in stigma toward MSM are important in reducing loneliness and hopefulness among MSM. Self-control links up the relationships between psychosocial problems and IGD and should be given special attention. Longitudinal studies are warranted to confirm the findings and test new mediations between loneliness/hopelessness and MSM with IGD.

## Introduction

Men who have sex with men (MSM) is a sexual minority group that is vulnerable to addictive behaviors [1]. For instance, high prevalence of substance use has commonly been reported among MSM [1]. According to the minority stress model, minority groups are more likely than their counterparts to encounter stress arising from prejudice and discrimination due to their minority status, which would trigger maladaptive stress responses leading to negative health outcomes [2]. Addictive behaviors (eg, substance use, gambling, and internet gaming) can be considered as potential maladaptive stress responses [3-5]. Thus, sexual minority stress experienced by MSM may drive them to adopt addictive behaviors as a means of maladaptive coping. Internet gaming disorder (IGD) is an important form of such potential maladaptive responses, as internet and social media use have globally become a part of daily life.

IGD has become a growing public health concern, as it is prevalent and was associated with many potential harms including depression and increased violence [6]. Its significance is reflected by the inclusion in the *International Classification of Diseases, 11th Revision* as a form of mental disorder [7]. High prevalence of IGD was found in adolescents and emerging adult populations [8,9]. Among emerging adults (university students) in mainland China, the prevalence was 9.9% to 14.8% [8]. Social media are frequently used by MSM for social interactions with peers and seeking sex partners [10]; internet gaming among MSM might sometimes be mixed with such purposes. Despite the significance and high risk of IGD due to their sexual minority status among MSM, no study has specifically looked at the prevalence of IGD among MSM, according to our literature review. Although a study reported higher prevalence of problematic gaming among nonheterosexual than heterosexual participants (26% vs 13%), it did not measure IGD specifically [11]. This study represents a novel exploration of the prevalence and factors of IGD among MSM who are emerging adults (university students) in mainland China.

MSM tend to have high levels of loneliness and hopelessness [12,13]. The positive associations between loneliness/hopelessness and IGD have been well established, both theoretically and empirically in general and student populations [8,14,15]. Understanding such associations among MSM is highly warranted. Theoretically, the minority stress model postulates that stressors arising from experiences of prejudice events, expectation of prejudice reactions, and disharmony between the MSM subculture and the mainstream culture would create loneliness and hopelessness among MSM [2]. Perceived and enacted stigma, lack of social support, and loss of friends and intimate partners due to HIV/AIDS were also associated with loneliness and hopelessness among MSM [2]. Furthermore, previous studies have shown that young MSM are prone to experience greater loneliness than their older counterparts [16,17]. A potential consequence is an elevated risk in developing IGD among more younger than older MSM. Furthermore, the preference for online social interaction model postulates that those experiencing psychosocial problems in real life (loneliness and hopelessness in this case) may use internet-related activities (internet gaming in this case) to compensate their unmet psychosocial needs (eg, self-worth), thus leading to a higher risk in developing specific internet use disorder (IGD in this case) [18].

The mechanism between loneliness/hopelessness and IGD among MSM would be informative for designing effective interventions but has not been studied. Self-control is a potential mediator between loneliness/hopelessness and IGD. Self-control has special relevance as MSM exhibited weaker self-control than non-MSM [19,20]. Theoretically, the deficient self-regulation model postulates that self-control mediates between stress and problematic internet use, as self-control may suppress negative emotions caused by stress [21]. Empirical studies have reported a negative association between loneliness/hopelessness and self-control [22,23], and self-control was negatively associated with IGD [24,25]. A study also reported significant mediation between loneliness and problematic gambling via self-control [26].

This study investigated the prevalence and factors (ie, loneliness, hopelessness, and self-control) of IGD among university students who were MSM in mainland China. It also investigated the mediation effect of self-control between loneliness/hopelessness and IGD. It was hypothesized that loneliness and hopelessness would be positively associated with IGD, loneliness and hopelessness would be negatively associated with self-control, and self-control would be negatively associated with IGD. The direct effects from loneliness and hopelessness to IGD were also tested in this study. The study has special relevance to the MSM population, as they tend to have higher levels of addictive behaviors and loneliness/hopelessness, and a lower level of self-control. The information would facilitate tailored IGD interventions targeting MSM.

## Methods

### Participants and Data Collection

An anonymous cross-sectional study was conducted among university students who were MSM in Chengdu, China during September and October 2018. Inclusion criteria included those aged ≥18 years, male full-time university students, self-reporting having sex with men in the past 6 months, and provision of informed consent. Convenience sampling was conducted. A local nongovernmental organization, Chengdu Tongle Health Counselling Service Center, trained and briefed 20 paid part-time outreach fieldworkers who were university students and MSM; these workers then recruited participants through their networks and by posting invitations on some online social networking sites frequently used by local MSM who are university students (eg, WeChat group and Blued). Interested prospective participants contacted the coordinators. After confirming the participants’ eligibility to join the study, the coordinators briefed the participants about the study and obtained their verbal informed consent. The coordinator would then send the participants a link to access the self-administered online questionnaire. It took about 20 minutes to complete. An incentive of ¥20 (about US $3) was given to participants who completed the web-based survey as a token of appreciation.

### Measurements

#### Background Information

Such information included year of study, study major, whether local residents of Chengdu, and average monthly expenditure.

#### Internet Gaming Disorder

IGD was assessed by using the 9-item *Diagnostic and Statistical Manual of Mental Disorders* (Fifth Edition; *DSM-5*) checklist [27], which assessed the presence of nine symptoms (preoccupation, withdrawal, tolerance, continuation of gaming despite problems, deception of gaming time, loss of control over gaming, prioritization over other activities, avoidance, significant loss due to gaming) in the past 12 months (response options: yes/no). IGD status was defined as those endorsing five or more of the nine symptoms. The Chinese version of the *DSM-5* checklist has been validated among Chinese adults [28,29] and applied to Chinese university students [8]. The Cronbach α was .87 in this study.

#### Loneliness

Loneliness was assessed using the 3-item short version of the UCLA Loneliness Scale [30], which assessed the feelings of lacking companionship, being left out, and being isolated. The items were rated by using 4-point Likert scales (1, never, to 4, often); higher scores indicated higher levels of loneliness. The Chinese version has been applied to the Chinese population [31]. The Cronbach α was .86 in this study.

#### Hopelessness

Hopelessness was assessed by using the 4-item Beck Hopelessness Scale [32]; its Chinese version has been validated among Chinese adults with satisfactory psychometric properties [33]. A sample item includes “My future seems dark to me.” The items were rated by using 6-point Likert scales (1, strongly disagree, to 6, strongly agree); higher scores indicated higher levels of hopelessness. The Cronbach α was .74 in this study.

#### Self-control

Self-control was assessed by using the 11-item Chinese Self-control Scale [34]. A sample item includes “It is hard for me to concentrate.” The items were rated by using 5-point Likert scales (1, almost never, to 5, always); higher scores indicated higher levels of self-control. The Cronbach α was .84 in this study.

### Sample Size Planning

Regarding sample size requirement, structural equation modeling (SEM) was used as the key statistical method; it requires a minimum of 10 cases per item/unobserved variable [35]. In our case, such a ratio was 16:1, which is acceptable. In addition, this study reported odds ratios for some associations. Assuming the prevalence of IGD ranged from 10% to 15%, a sample size of 305 would have the smallest detectable odds ratio of 1.61 to 1.76 (power of 0.80 and α of .05, 2-sided; logistic regression module in PASS 11.0, NCSS, LLC). The sample size is thus also adequate.

### Statistical Analysis

IGD was a binary dependent variable. Univariable logistic regression analysis was conducted to test the associations between background factors and IGD. Multivariable logistic regression analysis was conducted to test the associations between the three psychological factors (loneliness, hopelessness, and self-control) and IGD, adjusting for background factors. Crude odds ratio (ORc), adjusted odds ratio (ORa), and their respective 95% CIs were generated. SEM, using the weighted least squared mean and variance adjusted estimator, was conducted to test the potential mediation mechanisms that self-control would mediate between loneliness/hopelessness and IGD. Three latent variables were created: the two latent variables of loneliness and hopelessness were derived from the three and four original scale items, respectively, while the latent variable of self-control was derived from three randomly grouped parcels from the original 11 items (item parceling is recommended to reduce measurement errors of latent variables that are measured with relatively more items in SEM [36]). Satisfactory model fit index included χ^2^/*df*<5, comparative fit index (CFI) ≥0.90, Tucker-Lewis index (TLI) ≥0.90, and root mean square error of approximation (RMSEA) ≤0.80. Bootstrapping method (n=2000) was conducted to test the significance of the indirect effects whose 95% CI did not include zero, which would indicate significant indirect effects. SEM was conducted using Mplus 7.0 (Muthén & Muthén), while other analyses were conducted using SPSS 26.0 (IBM Corp). Statistical significance was defined as a 2-tailed *P*<.05.

### Ethics Approval

This study was approved by the Survey and Behavioral Research Ethics Committee of the Chinese University of Hong Kong (SBRE-21-0635).

## Results

### Participants’ Background

Of 305 participants, the mean age was 21.1 (SD 2.8, range 14-30) years. About 30.5% (n=93) were local residents of Chengdu, majored in arts subjects (n=88, 28.9%), and spent >¥2000 (about US $313) per month on average (n=84, 27.5%). About one-fourth were in their first year (n=69, 22.6%), second year (n=76, 24.9%), and third year (n=75, 24.6%). The prevalence of IGD was 12.8% (39/305; see Table 1). The mean scale scores were 37.8 (SD 8.4, range 14-54) for self-control, 6.6 (SD 2.8, range 3-12) for loneliness, and 9.1 (SD 3.8, range 4-24) for hopelessness.

**Table 1:** Descriptive statistics (N=305).

		Participants, n (%)
**Place of residence**		
	Chengdu	93 (30.5)
	Other cities	208 (68.2)
	No answer	4 (1.3)
**Study major**		
	Arts	88 (28.9)
	Business	40 (13.1)
	Science	135 (44.3)
	Others	22 (7.2)
	Not reported	20 (6.6)
**Year of study**		
	Year 1	36 (11.8)
	Year 2	69 (22.6)
	Year 3	76 (24.9)
	Year 4 or final year	75 (24.6)
	Not reported	49 (16.1)
**Average monthly expenditure (¥)**		
	≤2000 (about US $330)	212 (69.5)
	>2000	84 (27.5)
	Not reported	9 (3.0)
**Internet gaming disorder**		
	No	266 (87.2)
	Yes	39 (12.8)

### Background Factors of IGD

Univariable logistic regression analysis showed that the fourth year or final year students (ORc 0.23, 95% CI 0.06-0.86) were less likely than others to have IGD. The associations between sociodemographics (age, place of residence, study major, and average monthly expenditure) and IGD were nonsignificant among university students who were MSM (see Table 2).

**Table 2:** Associations between background factors and internet gaming disorder (N=305).

Independent variables			Internet gaming disorder, ORc^a^ (95% CI)
Age			0.92 (0.81-1.05)
**Place of residence**			
	Chengdu	1.0 (reference group)	
	Other cities	1.07 (0.50-2.26)	
	Not reported	7.56 (0.95-58.40)	
**Study major**			
	Arts	1.0 (reference group)	
	Business	1.11 (0.36-3.50)	
	Science	1.20 (0.53-2.74)	
	Others	1.23 (0.31-4.92)	
	Not reported	1.38 (0.34-5.54)	
**Year of study**			
	Year 1	1.0 (reference group)	
	Year 2	0.70 (0.24-2.03)	
	Year 3	0.78 (0.28-2.18)	
	Year 4 or final year	0.23 (0.06-0.86)*	
	Not reported	0.58 (0.18-1.90)	
**Average monthly expenditure (¥)**			
	≤2000 (about US $330)	1.0 (reference group)	
	>2000	1.03 (0.49-2.19)	
	Not reported	0.86 (0.10-7.12)	

^a^ORc: crude odds ratio.

*P<.05.

### Adjusted Associations Between Psychological Factors and IGD

Adjusted for background factors, multivariable logistic regression analysis showed that loneliness (ORa 1.29, 95% CI 1.14-1.47) and hopelessness (ORa 1.27, 95% CI 1.15-1.40) were both positively and significantly associated with IGD, while self-control was negatively associated with IGD (ORa 0.90, 95% CI 0.87-0.94; see Table 3).

**Table 3:** Adjusted associations between the three psychosocial variables and internet gaming disorder (N=305).

Independent variables^a^	Adjusted odds ratio (95% CI)
Loneliness	1.29 (1.14-1.47)***
Hopelessness	1.27 (1.15-1.40)***
Self-control	0.90 (0.87-0.94)***

^a^The models were adjusted for background variables including age, place of residence, study major, year of study, and average monthly expenditure.

***P<.001.

### Structural Equation Modeling

The model fit index of the measurement model of the three latent variables of loneliness, hopelessness, and self-control were satisfactory (χ^2^ / *df* = 70.960 / 32 = 2.22<5; *P*<.001; RMSEA 0.06; CFI 0.97; TLI 0.96). The factor loadings of the above three latent variables ranged from 0.38 to 0.91 (all *P*<.001). It indicates that the measurement model was suitable for conducting SEM.

**Figure 1:**
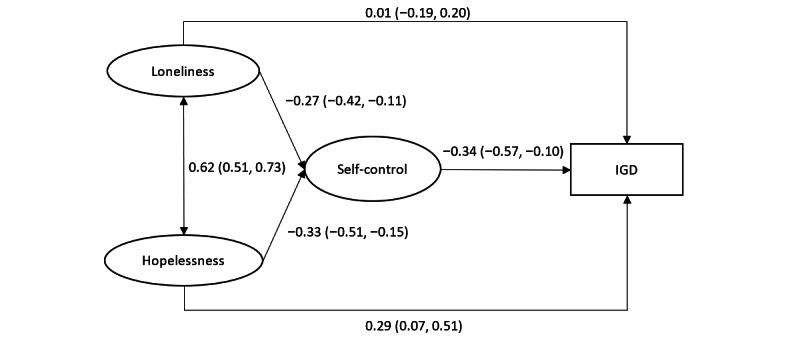
Structural equation modeling testing the mediation effect of self-control between loneliness/hopelessness and IGD (standardized coefficients were reported). IGD: internet gaming disorder.

Figure 1 presents the SEM results regarding the mediation effect of the latent variable of self-control between the latent variables of loneliness and hopelessness and IGD adjusting for the background factors. It gave a satisfactory model fit index (χ^2^ / *df* = 198.41 / 156 = 1.27<5; *P*=.01; RMSEA 0.03; CFI 0.93; TLI 0.91). First, the latent variable of loneliness was negatively associated with the latent variable of self-control (β=−0.27, 95% CI −0.42 to −0.11), which was also negatively associated with IGD (β=−0.34, 95% CI −0.57 to −0.10). The indirect effect of loneliness on IGD via self-control was significant (β=0.09, 95% CI 0.01-0.17); the direct effect of loneliness on IGD was nonsignificant (β=−0.01, 95% CI −0.19 to 0.20); a full mediation effect between loneliness and IGD via self-control was hence observed. Second, the latent variable of hopelessness was also negatively associated with the latent variable of self-control (β=−0.33, 95% CI −0.51 to −0.15), which was negatively associated with IGD as aforementioned. As both the direct effect (β=0.29, 95% CI 0.07-0.51) and indirect effect between hopelessness and IGD via self-control (β=0.11, 95% CI 0.02-0.21) were significant, a significant partial mediation effect between hopelessness and IGD via self-control (effect size of 28.0%) was observed.

## Discussion

This study observed a 13% (39/305) prevalence of IGD among Chinese MSM who were university students, which is relatively high and requires attention. It is unknown whether the prevalence would be higher than that of non-MSM who are university students as this study did not involve the non-MSM group and hence no comparison can be made. Future confirmation is warranted. Nonetheless, the high prevalence deserves attention and warrants interventions, as IGD was associated with mental problems such as depression [37] and addictive behaviors such as substance use [38] that may also increase the risk of HIV infection [1,39], which is a serious concern among Chinese MSM who were university students [40].

Year of study was the only background variable significantly associated with IGD. Year 4 students showed lower prevalence of IGD than other students. Previous studies reported similar results that lower grades (eg, first year) were associated with higher risk of addictive behaviors (eg, substance use) [41]. It is plausible that year 4 students might have less time for internet gaming as they were heavily engaged in job-hunting and graduation arrangements thus were at a lower risk of IGD.

As expected, loneliness and hopelessness were both positively associated with IGD in this study, which corroborates findings of other studies [8,14,15]. It also supports the Preference for Online Social Interaction (POSI) model claiming that psychosocial problems such as loneliness and hopelessness may become an inner drive that would increase the risk of problematic internet gaming [18]. The findings have special implications as previous studies found that MSM were at greater risk of other addictive behaviors (eg, drug use and smoking) than others [3,4]. Furthermore, MSM are in general vulnerable to loneliness and hopelessness due to potential discrimination and stigma related to their sexual identity [2]. The trend might lead to higher prevalence of IGD in MSM who are university students than their counterparts, which needs to be investigated in future studies. Notably, this study was conducted prior to the COVID-19 pandemic. A study reported that loneliness showed a stronger correlation with IGD during the COVID-19 pandemic [42]. Future research should study if this is true for this study population.

A more acceptable social environment is also warranted to reduce risk factors of IGD such as loneliness and hopelessness. In agreement with the socioecological model of health [43], the findings suggest that structural factors such as public discrimination toward MSM are important to influence factors of IGD and might hence increase prevalence of IGD among MSM who are university students. The difficulty in developing same-sex families might also contribute to stronger hopelessness. Same-sex marriage is not legally accepted in China and in many parts of the world. MSM might hence find the hope for establishing a family difficult to fulfill. To prevent IGD among MSM who are university students, according to the findings, interventions may consider reducing loneliness and hopelessness. Although such interventions are available, few of them target sexual minority groups. As an exception, a small one-armed pilot psychoeducation intervention reported mild reduction in the level of loneliness over a 3-month period among MSM [44]. As the reasons of loneliness and hopelessness might partially be attributed to sexual minority experiences, tailored individual-level interventions tackling self-stigma among MSM might be useful. For instance, Moreno’s therapeutic model used sociometry, psychodrama, and group psychotherapy to reduce shame and internalized homophobia and increase social support among MSM living with HIV [45].

Supporting one of the hypotheses of this study, self-control was negatively associated with IGD. The finding was supported by previous theoretical and empirical studies [21,23]. Those with strong self-control might deliberately resist the impulse of playing internet games and find it easier to stop playing [46,47]. Another novel finding of this study revealed that self-control fully mediated between loneliness and IGD. This finding agrees with the deficient self-regulation model that mental distress (loneliness and hopelessness in this case) would diminish self-control, which would increase the risk of IGD [21]. The full mediation between loneliness and IGD implies that the negative impact of loneliness on IGD could be fully explained by reduction in self-control. The significant partial mediation between hopelessness and IGD implies that other potential mediators might exist but had not been included in this study. For instance, hopelessness might lead to depression and adoption of negative coping styles that would increase IGD [5,6,48]. Importantly, the significant mediations suggest that modification of self-control would directly reduce IGD and indirectly alleviate the harmful impacts of loneliness and hopelessness on IGD. In the literature, self-control could be strengthened via trainable strategies such as ways to avoid temptations (eg, healthy distraction), plans made ahead of occurrence of temptations, meditation, and self-reminders about the consequences [49-52].

This study has several limitations. First, due to the cross-sectional study design, no causal and temporal inferences can be claimed. Second, reporting bias (eg, recall bias and social desirability bias) may exist and result in underreported prevalence of IGD and an inflated level of self-control. Third, selection bias may exist; the characteristics between MSM participants and nonparticipants might differ but could not be compared. Fourth, the participating universities were conveniently selected in the studied city; generalization of the results to other regions in and outside China should be cautiously made. Fifth, this study did not investigate other potential determinants of IGD that were closely related to the MSM status (eg, sexual orientation, sexual role, and sexual behaviors). Sixth, other potential mediators of the association between hopelessness and IGD may exist but had not been investigated; some of these factors/mediators of IGD may have special relevance to MSM, such as internalized stigma and cognitive bias [2,53]. Last, a comparison group of non-MSM who are university students had not been included in this study; it is hence unknown whether the levels of IGD, loneliness, hopelessness, and self-control of the participants were different between MSM and non-MSM who are university students. Comparative studies would be informative.

In conclusion, this study observed relatively high prevalence of IGD among MSM who are university students in China, which was significantly associated with loneliness, hopelessness, and self-control. Interventions preventing IGD among MSM who are emerging adults might hence need to simultaneously improve loneliness, hopelessness, and self-control. A novel finding of this study was that self-control significantly (and fully or partially) mediated between loneliness/hopelessness and IGD, respectively. The significant mediation effects highlight the importance of self-control apart from its well-studied direct effect on IGD. It suggests that self-control links up the relationships between commonly found psychological problems (eg, loneliness and hopelessness) and IGD among MSM. Future longitudinal and intervention studies are needed to verify the above findings among MSM and other sexual minority groups.

## Abbreviations

CFI: comparative fit index
DSM-5: Diagnostic and Statistical Manual of Mental Disorders (Fifth Edition)
IGD: internet gaming disorder
MSM: men who have sex with men
ORa: adjusted odds ratio
ORc: crude odds ratio
RMSEA: root mean square error of approximation
SEM: structural equation modeling
TLI: Tucker-Lewis index
